# Anomaly Detection Algorithm for Real-World Data and Evidence in Clinical Research: Implementation, Evaluation, and Validation Study

**DOI:** 10.2196/27172

**Published:** 2021-05-07

**Authors:** Vendula Churová, Roman Vyškovský, Kateřina Maršálová, David Kudláček, Daniel Schwarz

**Affiliations:** 1 Faculty of Medicine Masaryk University Brno Czech Republic; 2 Institute of Biostatistics and Analyses, Ltd Brno Czech Republic

**Keywords:** clinical research data, real-world evidence, registry database, data quality, EDC system, anomaly detection

## Abstract

**Background:**

Statistical analysis, which has become an integral part of evidence-based medicine, relies heavily on data quality that is of critical importance in modern clinical research. Input data are not only at risk of being falsified or fabricated, but also at risk of being mishandled by investigators.

**Objective:**

The urgent need to assure the highest data quality possible has led to the implementation of various auditing strategies designed to monitor clinical trials and detect errors of different origin that frequently occur in the field. The objective of this study was to describe a machine learning–based algorithm to detect anomalous patterns in data created as a consequence of carelessness, systematic error, or intentionally by entering fabricated values.

**Methods:**

A particular electronic data capture (EDC) system, which is used for data management in clinical registries, is presented including its architecture and data structure. This EDC system features an algorithm based on machine learning designed to detect anomalous patterns in quantitative data. The detection algorithm combines clustering with a series of 7 distance metrics that serve to determine the strength of an anomaly. For the detection process, the thresholds and combinations of the metrics were used and the detection performance was evaluated and validated in the experiments involving simulated anomalous data and real-world data.

**Results:**

Five different clinical registries related to neuroscience were presented—all of them running in the given EDC system. Two of the registries were selected for the evaluation experiments and served also to validate the detection performance on an independent data set. The best performing combination of the distance metrics was that of Canberra, Manhattan, and Mahalanobis, whereas Cosine and Chebyshev metrics had been excluded from further analysis due to the lowest performance when used as single distance metric–based classifiers.

**Conclusions:**

The experimental results demonstrate that the algorithm is universal in nature, and as such may be implemented in other EDC systems, and is capable of anomalous data detection with a sensitivity exceeding 85%.

## Introduction

Adherence to principles of evidence-based medicine has become the norm in the present-day clinical practice. Such principles include establishing proper guidelines built upon evidence derived from the best available clinical research. Therefore, high quality of input data is of utmost importance, because otherwise biased evidence may be generated, possibly resulting in harmful health decisions.

Clinical registries, defined as a systematic collection of clearly defined set of health and demographic data gathered from patients with specific health characteristics, represent one of many data sources available in health care [1]. The impact of clinical registries on quality of patient care taking account of a clinical research perspective is reviewed in [2], where monitoring health care delivery patterns and compliance with the evidence-based guidelines are also examined. The real-world data (RWD) collected in these registries may, in the context of postmarket research, provide much needed answers to questions unaddressed by existing randomized controlled trials. As patient populations participating in clinical trials are frequently low in numbers and rather homogenous and highly specific, further usage of such obtained data sets for the purpose of predicting medical treatment outcomes or future performance in the real-world, uncontrolled conditions has proved to be difficult [3].

The efficiency of data analysis is heavily dependent on data quality that has the potential to impact clinical research outcomes in both controlled clinical trials and postmarket surveillance practice represented mostly by noninterventional, observational studies and clinical registries. Data quality–related issues, such as high proportion of missing or inaccurate data, bring uncertainty to the final analytics, slow workflows, generate extra work, and thus increase research costs. A review and a generic framework for data quality in medical registries are given in [4], including some types and percentages of various data errors in a case study. In another scoping review [5], which focused on trauma registries, a call for standardization of classification, measurement, and improvement of data quality can be found. In order to mitigate data quality issues, various auditing techniques and monitoring strategies have been put in place (see the review in [6]). Besides extensive monitoring approaches including on-site visits and exhaustive source data verification, other effective risk-based monitoring methods have recently been implemented in the field of data quality assurance. These reduce monitoring costs by utilizing advanced statistical tools capable of identifying medical centers or clinics with atypical data patterns which might signify a quality problem [7]. The statistical concepts underpinning the central statistical monitoring (CSM) designed to detect fraud, that is, fabrication or falsification of data, were proposed 2 decades ago. The incidence of data fraud in clinical research is considered to be relatively low, yet difficult to estimate accurately [8].

Conventional data collection in clinical research involves recording data in paper case report forms (CRFs), followed by a double entry in a relational database. Continuous technological advancements in computer science, life sciences, and health care have given rise to the electronic data capture (EDC) systems, which have proved to be a more efficient [9] and also a cheaper [10] alternative to the paper data capture. EDC systems enable investigators to enter data directly into electronic CRFs (eCRFs) and study coordinators to oversee and control them in real time [11-13] even in multicenter research studies. EDC systems have become predominant because they are not only time- and cost-effective, but also contribute to quality assurance, as they allow data access to be controlled and all changes made to them using audit trail features to be traced. Moreover, they perform automatic edit checks designed to prevent invalid data from being entered [14] into a clinical registry, which is, however, hardly possible to be ruled out completely. When multiple variables need to be constrained by edit checks, the validation procedures, designed by data managers, may become too complicated and prone to error. The alert messages resulting from such complex edit checks may be unintelligible to clinical investigators, who still need to understand their factual content, as the validation procedures form an integral part of the eCRF.

Thus, there is still great potential for further improvements in ensuring high quality of data with the use of EDC systems. Integration with the aforementioned risk-based monitoring tools, such as CSM employing various outlier detection techniques, represents another automated approach to quality control. The review in [15] divides the outlier detection techniques, which have been used for data assurance in health care databases, into several categories: statistical, clustering, classification, nearest neighbor, and mixture models. It reveals that the statistical techniques are used frequently, whereas the other ones associated with data science and data mining are still little used in this context.

The viewpoint presented in [16] questions the benefits of a particular CSM technique which classified clinical sites as outlying based on the data inconsistency score calculated from thousands of statistical tests in a particular multicenter, postmarketing trial, and therefore dismissed the idea that trials could be conducted at lower costs.

This paper describes an algorithm based on machine learning designed to detect anomalous patterns in data created as a consequence of carelessness, systematic error, or intentionally by entering fabricated values. It focuses on the main concepts defining the anomaly detection algorithm and presents a particular EDC system demonstrating its successful implementation. The data sets collected by this system have been used in a number of clinical registries and serve here for pilot testing and calculations of anomaly detection rates. It is important to note that by anomalous data or an anomaly we understand an observation that does not conform to normally gathered data, where an observation refers to a single patient data record entering the detection algorithm.

## Methods

In order to fully implement an anomaly detection algorithm, which is data structure dependent, having an EDC system in place is essential. A thorough description representing such a system, including its architecture and data structure, is presented in this section.

### EDC System and Its Data Structure

The EDC system utilized in this study referred to as the Clade-IS (Clinical Data Warehousing Information System) is a robust, modular, web-based software for data management and clinical trial management. It contains a huge amount of RWD from many clinical specialties, including neurology and psychiatry, that are readily available to be used for experimentation. The authors of this paper are engineers, data scientists, computer scientists, and data managers affiliated with the contract research institution where this EDC system has been developed and so they have a very good understanding of its data structures.

**Figure 1:**
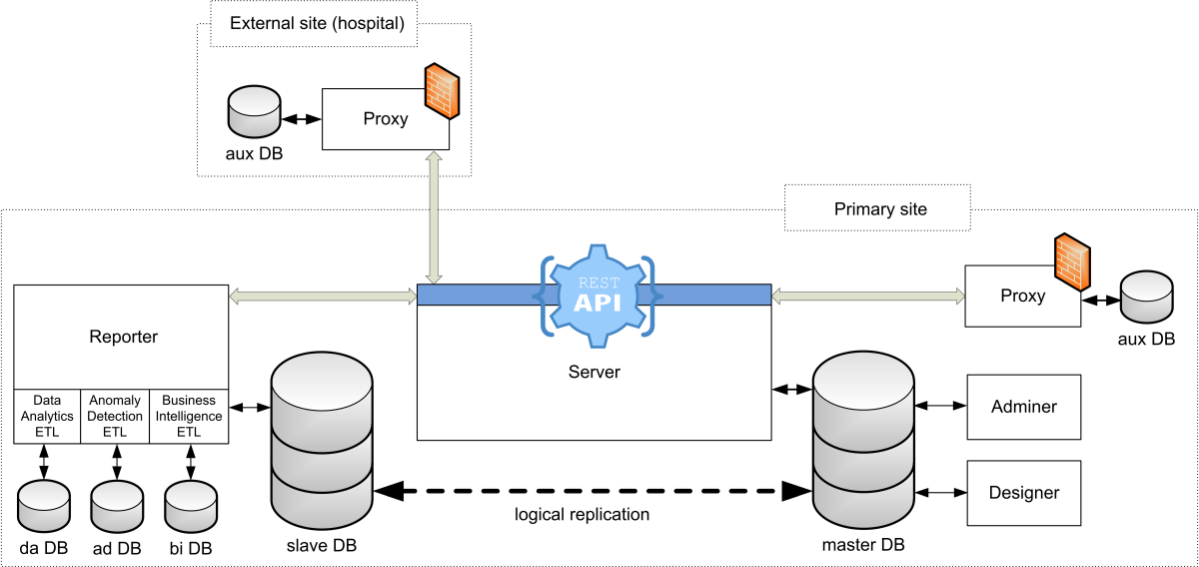
Architecture of the Clinical Data Warehousing Information System (Clade-IS) components and databases. The Server provides a representational state transfer application interface (REST API) for most operations including data storage. The Proxy represents a forwarded interface that transfers the user’s activity to the Server. The Proxy can be optionally decentralized into a hospital or to another research facility. The Adminer and the Designer are used for configuring registry-specific permissions, designing electronic case report forms (eCRFs) and also for building and generating forms that are accessible to authenticated and authorized users. The Reporter is based on extract–transform–load (ETL) processes and serves for analytical and reporting purposes.

The system is composed of 5 mutually communicating components: proxy, server, adminer, designer, and reporter; see the architecture in Figure 1. The proxy, representing the user interface, propagates the user’s activity, defined by requests made through a REST API (representational state transfer application interface) to the server, where the requests are processed. The server also stores and accesses registry data in a relational database and maintains data integrity. For example, the consistency and accuracy of data must be ensured throughout the transition between the components, as the format of the data varies according to its intended use, from its input, through storage, to extraction and reporting. It also ensures compliance with data access rules, which can be configured via users, groups, roles, and form statuses in the adminer. The next component, called the designer, represents a comprehensive form builder used by data managers to design eCRFs.

Finally, the reporter, serving as a toolkit for data analysts and data scientists, is a component based on the ETL (extract–transform–load) processes that facilitate data export and business intelligence. Besides the master database, which primarily serves for data storage operations, there are 4 other databases that the aforesaid components use for the following purposes: (1) the slave database is a logical replica of the master one performing all data extraction operations; (2) the proxy auxiliary database stores personal data gathered in research projects and studies outside the central repository in case that the centralized deployment of the Clade-IS is no longer possible under the General Data Protection Regulation (GDPR) on digital data; (3) the reporter-*ad* database serves for data export purposes; and (4) the reporter-*bi* database is used for business intelligence reporting.

**Figure 2:**
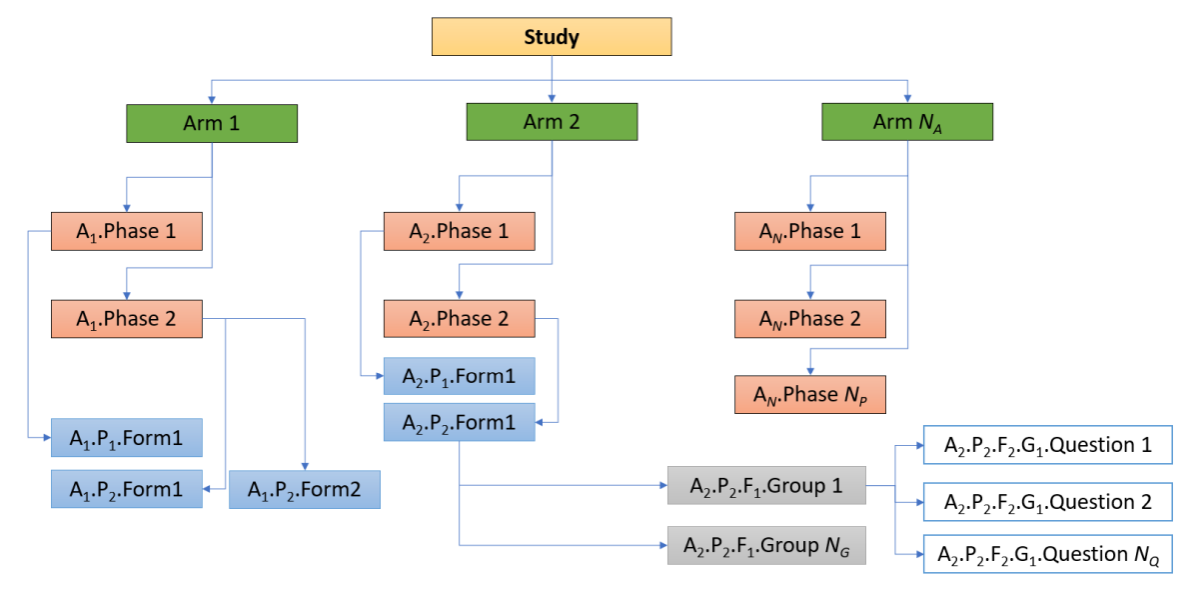
An example illustrating a structure of entities (arms, phases, forms, question groups) and attributes (questions) used for structuring electronic case report forms (eCRFs) in the Clinical Data Warehousing Information System (Clade-IS). Questions are logically grouped into question groups (eg, Demography question group, Comorbidities question group, etc), a form is composed of question groups (eg, Diagnosis form, Treatment form, etc), forms are grouped into phases (eg, Hospitalization forms phase, Follow-up forms phase, Quality of life forms phase, etc), and phases are grouped into arms which may represent different sub-populations of subjects in a study or a registry (eg, subjects diagnosed with affective disorders, schizophrenia, schizoaffective disorders and control subjects).

The primary databases (master and slave) integrated into the Clade-IS are based on the entity–attribute–value (EAV) model, also known as the vertical database model, which is able to efficiently encode entities with sparse features. Such a functionality is directly applicable to clinical registries, as they typically contain plenty of available attributes describing an entity, but the number of attributes with assigned values is, once the data has been entered, rather low. The following data structures are used to build an eCRF: arm–phase–form–question group–question–answer, where a question–answer pair represents an attribute–value pair, respectively. The other structures represent entities in the EAV data model. Figure 2 serves to explain the meaning of the entities. The eCRF data are stored in JSON format; for instance, a single-answer question (Q10, Patient’s age at diagnosis) is represented as *“Q10”:{“value”:63,“state”:“done”}* and stored in a single cell in the database; see Multimedia Appendices 1 and 2 for data examples. The other database schemas differ depending on their specific purpose.

### Anomaly Detection Algorithm

**Figure 3:**
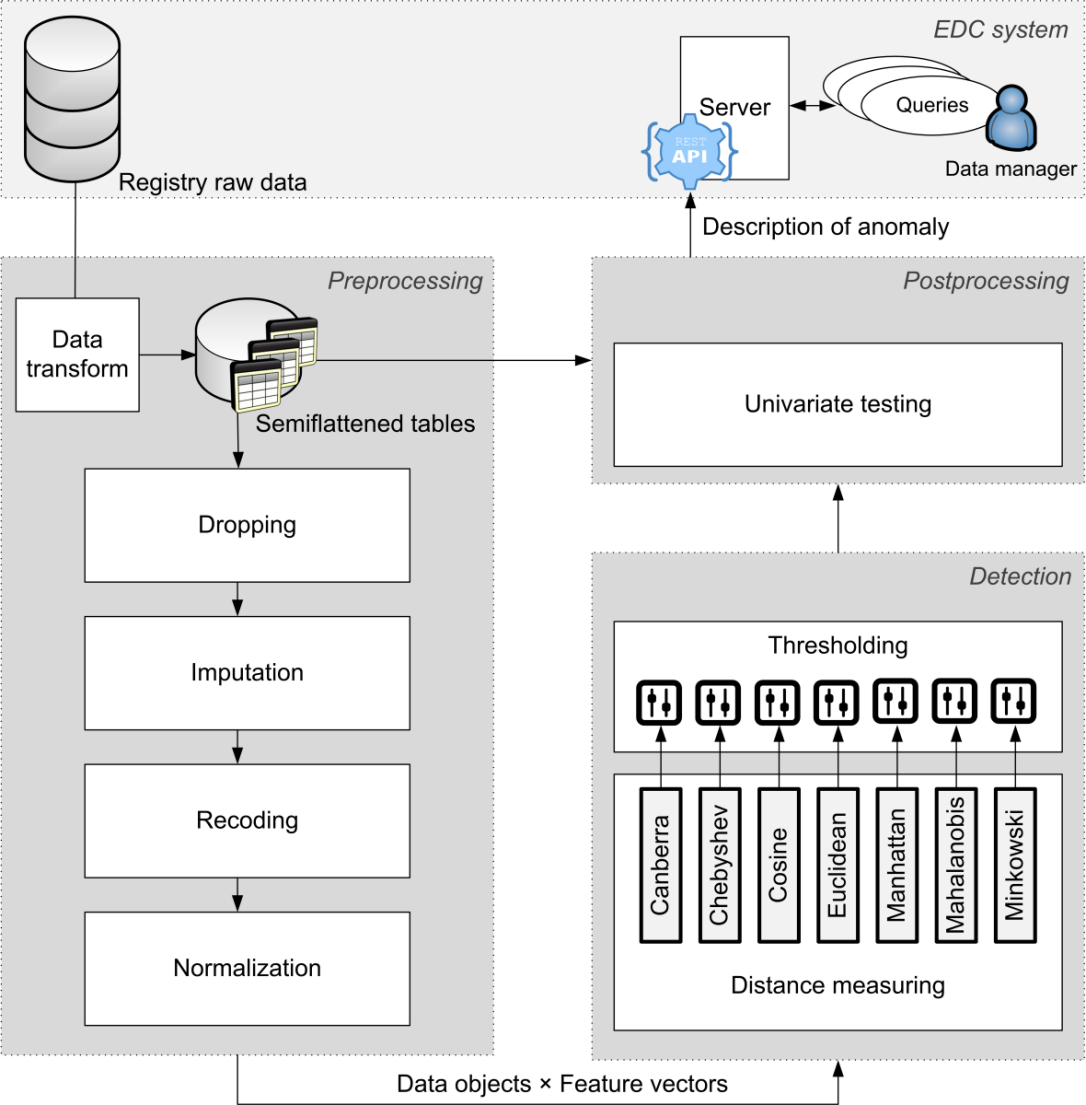
A scheme illustrating the anomaly detection algorithm and its links to the electronic data capture (EDC) system. The algorithm transforms registry raw data into semi-flattened tables which contain only meaningful combinations of variables in rows. The tables are preprocessed in four consecutive steps resulting in feature vectors from which one single centroid is computed. The distance between all data objects (feature vectors) and the centroid is measured using seven different distance metrics. The number of threshold-exceeding distances shows the strength of evidence of an anomaly. All anomalies are then subjected to post-hoc univariate tests to identify potentially problematic variables in the description of automatically generated electronic queries intended to be processed by data managers.

Anomalous data are identified by a scheduled script, built in the reporter component, that connects to the reporter-ad database, where it stores and accesses data in its own auxiliary tables. The main steps defining the detection algorithm are described in Figure 3.

**Figure 4:**
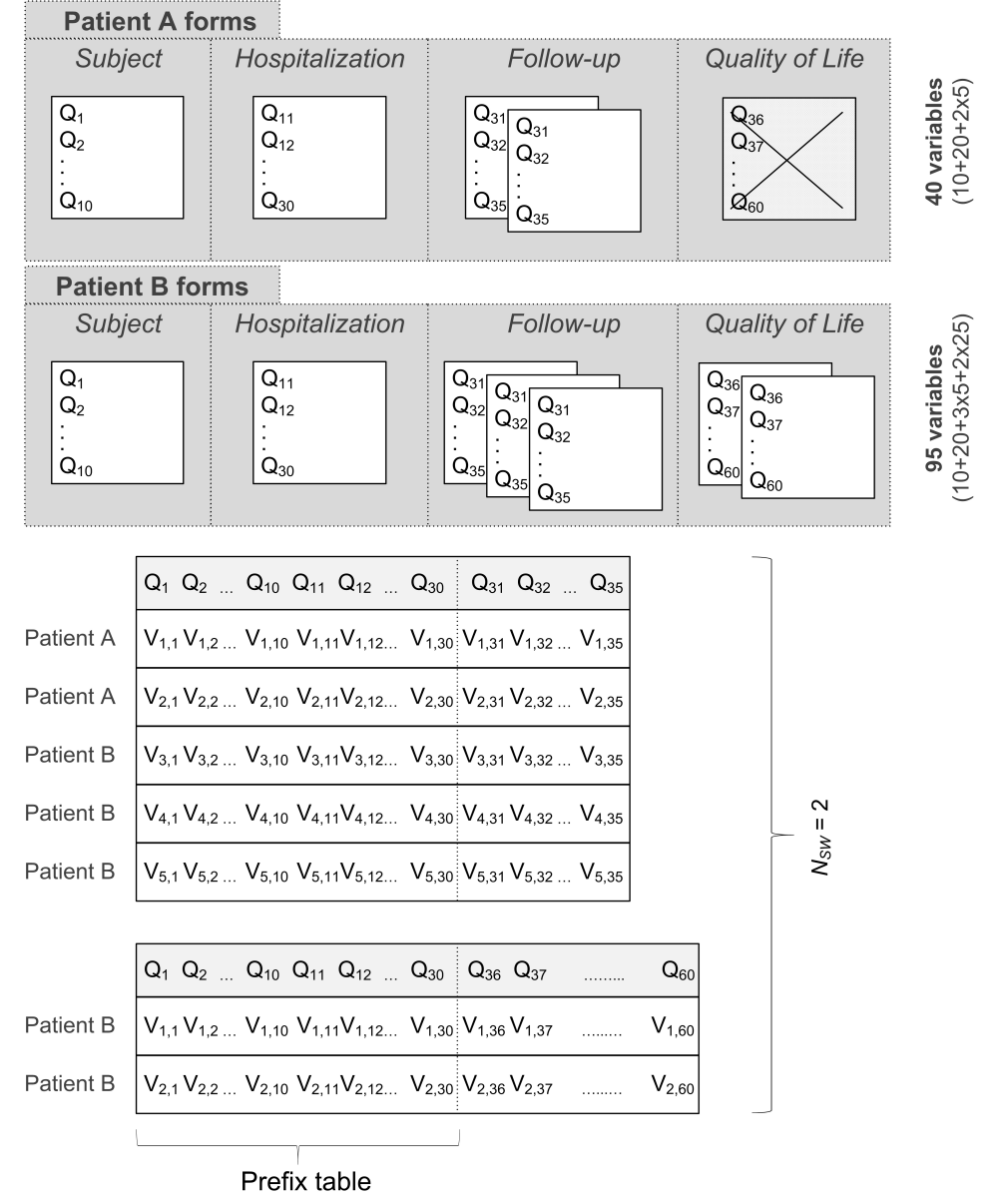
The concept of semi-flattened tables demonstrated on two patients‘ data. Two semi-flattened tables (N_sw_=2) result here from two different repeating forms: Follow-up and Quality of Life. The other two forms: Subject and Hospitalization exist only in one single instance per patient, and thus all their variables (Q1, Q2,…,Q30) put together a prefix table. Multiple existence of the semi-flattened tables occurs with electronic case report forms (eCRFs) that allow multiple forms creation. Each instance of a repeating form is appended to the prefix table. This concept with semi-flattened tables assures that all values aligned in a column are related to the same variable.

The multidimensional nature of the detection algorithm requires that all eCRF questions be merged into 1 flat-wide table, where the rows represent the patients and the columns represent the individual variables (attributes) collected from all forms across the eCRF structure. In order for a single flat-wide table to be considered an appropriate analytical data set, each patient would need to be linked to any of the forms in a 1:1 relationship. In most registries, however, a patient is linked to his/her forms in a 1:*N* relationship, where *N* usually differs between patients. For instance, patient A records may comprise 1 patient form, 1 hospitalization form, 2 follow-up forms but, say, no quality-of-life investigation form, whereas patient B records may comprise 1 patient form, 1 hospitalization form, 3 follow-up forms, and 2 quality-of-life investigation forms. Merging all forms into a flat-wide table would result in misalignment of variables in columns. Even eCRFs with an extremely rigid structure and predefined number of form instances per patient may still produce meaningless column combinations in terms of temporal context of a patient’s condition. To help overcome this problem, a concept of semiflattened tables is introduced here (Figure 4). The semiflattened tables consist of a “prefix” table, which is created by serializing all forms, allowing only a single instance to be run and 1 merged form that can be instantiated multiple times. This explains how *N_sw_* semiflattened tables are created, where *N_sw_* is the total number of all forms allowing multiple instances per patient. Therefore, the detection algorithm has to be run *N_sw_* +1 times for the prefix table and for each semiflattened table independently. The rows in both the prefix and the semiflattened table contain variables of the following data types: string, text, integer, float, date, datetime, time, Boolean, and categorical variables. Because only numerical data are subjected to further analysis, data tables need to be preprocessed. There are 4 preprocessing steps in the algorithm: dropping, imputation, recoding, and normalization. First, variables for which the amount of missing data exceeds a preset percentage are dropped (excluded) from the table. Besides, all variables of string and text data types are dropped, as they represent only unimportant notes and comments irrelevant for this study. The remaining variables, which still have some missing values, are imputed using median that is calculated for each variable separately. In the next step, the non-numerical variables are recoded to numerical ones. The variables of date, time, and datetime data types are recoded to numerical values representing the number of seconds since 1.1.1600 00:00:01. The Boolean variables and the categorical variables are recoded in a way that each unique data item represents a different integer value (eg, “Female” 1 and “Male” 0). The ascending integer values are assigned according to a frequency of occurrence of unique data. The numerical variables holding integer and float data types do not require any recoding.

In the last preprocessing step, the data must be normalized because the variables may vary in orders of magnitude or units of measurement. At the very end of the preprocessing phase, the data table looks as follows: each row represents an observation with columns representing variables with acceptable proportion of missing data that are recoded to their numerical representations and subsequently (min–max) normalized to produce values between 0 and 1; see the example data before and after preprocessing in Table 1.

Once the fully automatic preprocessing phase is complete, the anomalous data are classified similarly to how it is done in [15,17] using well-known clustering-based outlier detection techniques that also regard outliers as data objects not located in clusters of a data set. Here, only 1 cluster containing all data objects is created. Each object is described by a feature vector that takes the form of a row obtained from the preprocessed data table. The distance between a potential outlier and the cluster centroid is measured using 7 different distance metrics: Canberra (CAN), Chebyshev (CHEB), cosine (COS), Euclidean (EUC), Manhattan (MAN), Mahalanobis (MAH), and Minkowski (MINK). The aim of the proposed algorithm is not to perform a cluster analysis as the well-known k-means algorithm normally does. Instead, it seeks to find all data objects whose distance from a centroid is greater than a threshold differentiating anomalous records from the normal ones. The distance thresholds are calculated individually for each metric in 2 ways: (1) with a predefined percentile and (2) using the IQR rule, which sets the upper bound of the IQR multiplied by 1.5 and added to the third quartile. Data objects are identified as anomalous when at least one distance metric exceeds the minimum of both thresholds. With the predefined percentile, if the value is lower than the threshold specified by the IQR rule, the detection sensitivity can be increased, but usually at the expense of specificity. The strength of evidence of an anomaly is determined by the number of threshold-exceeding distance metrics.

The algorithm produces a table containing all detected anomalies represented by a patient identifier, the strength of evidence, and a list of potentially problematic variables identified using post-hoc univariate tests, which are different for normally and non-normally distributed variables. Afterward, a scheduled handler operating inside the reporter generates automatic queries which are of great concern to data managers, who are usually responsible for addressing them over the course of a study or a registry monitoring process.

**Table 1:** Example data before and after preprocessing. The index in rows represents a unique patient identifier. The column headings represent unique question identifiers—variable names encoding location in the study structure. The variables with a missing data rate of more than 20% were dropped. The other variables, which have an acceptable proportion of missing data, were imputed with median values. The data were subsequently recoded depending on the variable data type, and normalized to produce values in the interval [0, 1].^a^

Index		A0.P0.F2.G2.Q1	A0.P0.F2.G2.Q3	A1.P1.F7.G44.Q672	A1.P1.F7.G35.Q1443	A1.P1.F3.G3.Q6
**Before preprocessing**						
	0001437	1947-01-23	Female	Yes	4	64
	0001437	1947-01-23	Female	Yes	4	64
	0001437	1947-01-23	Female	Yes	4	64
	0001333	1941-06-24	Female	Yes	2	68
	0001333	1941-06-24	Female	Yes	2	68
	0001479	1948-11-03	Male	None	2	57
	0001513	1950-03-26	Male	Yes	1	59
**After preprocessing**						
	0001437	0.340432	1	0	1	0.657143
	0001437	0.340432	1	0	1	0.657143
	0001437	0.340432	1	0	1	0.657143
	0001333	0.258807	1	0	0.5	0.714286
	0001333	0.258807	1	0	0.5	0.714286
	0001479	0.366453	0	1	0.5	0.557143
	0001513	0.366453	0	0	0.25	0.585714

^a^A: study arm; P: study phase, where the related form is located at; F: form, where the question is located at; G: question group; Q: question.

### Simulation of Data Anomalies and Performance Evaluation

In the context of this study, evaluation refers to an exploratory analysis designed to establish quantitative characteristics of anomaly detection performance of the algorithm built into the aforementioned EDC system. The performance evaluation is carried out using simulated anomalous data, which need to be artificially generated inside an anomaly-free data set, in order to obtain the ground-truth knowledge.

The simulated anomalies, that are generated in a wide format table, are subsequently preprocessed by dropping, imputation, and recoding, but not by normalization. First, a small percentage (1% by default) of all cells in the table being preprocessed is set as the number of values *N_c_* intended to be changed. Second, a random number of patients is set as the number of anomalous data objects *N_s_* intended to be generated. The ratio *N_v_* = *N_c_*/*N_s_* gives an approximate number of variables whose values need to be changed in order to transform a normal data object into an anomalous one. These changes are performed only on variables of the following data types: integer, float, date, time, and datetime. The values of normally distributed variables are transformed to a mean (6σ), whereas the values of non-normally distributed variables are transformed to random numbers from an interval formed by rather unusual values having a frequency of occurrence lower than 10% in a particular variable. Afterward, the Shapiro–Wilk test, able to discriminate between normal and non-normal distributions, is run. Every time an anomaly occurs, the automatic edit checks built into a given registry are triggered, assuring that the newly generated, anomalous data undergo the same validation procedures as if having been entered by a human investigator. At the end of the simulation, all the generated anomalous data objects are identified in terms of their position in the data table, either as original or as changed values.

Performance evaluation of the detection algorithm is carried out in 2 phases: (1) setting the best thresholds for each distance metric and (2) finding the best combination of the distance metrics. In the first phase, the receiver operating characteristic (ROC) curves are calculated for each individual distance metric by varying the threshold percentile value. The best threshold is then selected based on the *C*_1_ criterion, which maximizes overall accuracy and Youden index [18], whereas the distance of the corresponding point on the ROC curve from the upper left corner *ULC_dist* is minimized:

*C_1_ = normalized (accuracy)^2^ + normalized (Youden index)^2^ – normalized (ULC_dist)^2^* **(1)**

where all 3 members of (1) are normalized to the interval (0, 1). All possible combinations of the distance metrics with the set threshold are then tested and the best one is determined by the *C*_2_ criterion which is based on balanced accuracy but favors sensitivity over specificity:

*C_2_ = balanced_accuracy + sensitivity = (TPR+TNR)/2 + TPR = (3TPR+TNR)/2* **(2)**

where TPR and TNR stand for true positive rate and true negative rate, respectively.

### Validation

In this study, validation refers primarily to repeatability verification which is performed as follows: all data from 2 different registries were subjected to expert review. As no problems were reported, these data sets could be used for evaluation and validation purposes. Once the detection algorithm is fully specified by the thresholds and the best combination of the distance metrics is identified by applying the 2-stage evaluation process to the first registry data, an independent data set from the second registry is used to validate the detection performance.

## Results

### EDC System Deployment

To date, the Clade-IS has been implemented in hundreds of clinical centers where it serves numerous research studies, mostly clinical registries and other RWD projects. Therefore, this EDC system contains millions of authentic records of different origin. Such a huge set of RWD made it possible to carry out anomaly detection using the designed detection algorithm whose performance was subsequently validated.

Five neuroscience-related registries were utilized here to investigate the possibility of deploying and using the aforementioned algorithm for automatic detection of anomalous data. The registries significantly differed in scope, that is, in research objectives, complexity of the eCRFs, duration, and also the number of patients involved (Table 2). While 2 out of 5 registries are sponsored by Masaryk University, 3 remaining registries belong to the neuromuscular section of the Czech Neurological Society, which did not allow their identification. For the sake of consistency, the names of all 5 registries are anonymized here.

Registry number 1 collects data on patients with myasthenia gravis, a rare, autoimmune disease affecting neuromuscular transmission. The registry serves to gather comprehensive information from as many patients as possible, covering the whole course of the disease and the response to treatment, in order to enhance development of new therapies and improve patient care. Registry number 2 collects data on patients diagnosed with any of the following neuromuscular diseases: Duchenne and Becker muscular dystrophy, spinal muscular atrophy, myotonic dystrophy, and facioscapulohumeral muscular dystrophy. The aim of the registry is to gather comprehensive information from as many patients with causal genetic defects as possible and thus to contribute to development of new treatments. Registry number 3 collects data on patients with spastic paresis caused by acquired brain injuries including a craniocerebral trauma, cerebral palsy, and central stroke. The aim of the registry is to develop visual analytics over the collected data to enhance decision-making processes related to physical and medical therapy at an individual patient level. Registry number 4 represents a longitudinal monitoring of patients with a cognitive impairment in the depressive phase of various affective disorders. The aim of the registry is to evaluate the diagnostic and prognostic potential of changes produced in brain morphology and function in patients with cognitive impairments and to investigate their impact on quality of a patient’s life and social functioning. Registry number 5 represents a 5-year, noninterventional, prospective follow-up study involving patients in the first episode of schizophrenia. The study aims to evaluate patients’ psychosocial needs in the early stages of the disease and also examine the effects of psychosocial interventions.

**Table 2:** Summary data presenting 5 neuroscience-related clinical registries powered by the Clade-IS^a^ utilized to investigate the possibility of performing automatic detection of anomalous data.

Quantitative characteristic × registry characteristic	Registry number 1	Registry number 2	Registry number 3	Registry number 4	Registry number 5
Forms	4763	9372	13,711	214	67
Patients	1150	1649	405	33	29
Investigators	26	63	15	19	8
Sites	9	14	1	1	1
Years of study	5	9	1	4	5

^a^Clade-IS: Clinical Data Warehousing Information System

### Anomaly Detection Algorithm—Evaluation and Validation

The performance of the detection algorithm was evaluated using the data set extracted from Registry number 3 and then validated using the data set extracted from Registry number 5. The simulated anomalies were generated for each data set separately using the procedure described in the next section. The default number of cells to be changed was set to 1%, that is, 22 normal data objects were transformed to anomalous in the evaluation data set (Registry number 3) and 7 normal data objects were transformed to anomalous in the independent data set (Registry number 5).

**Figure 5:**
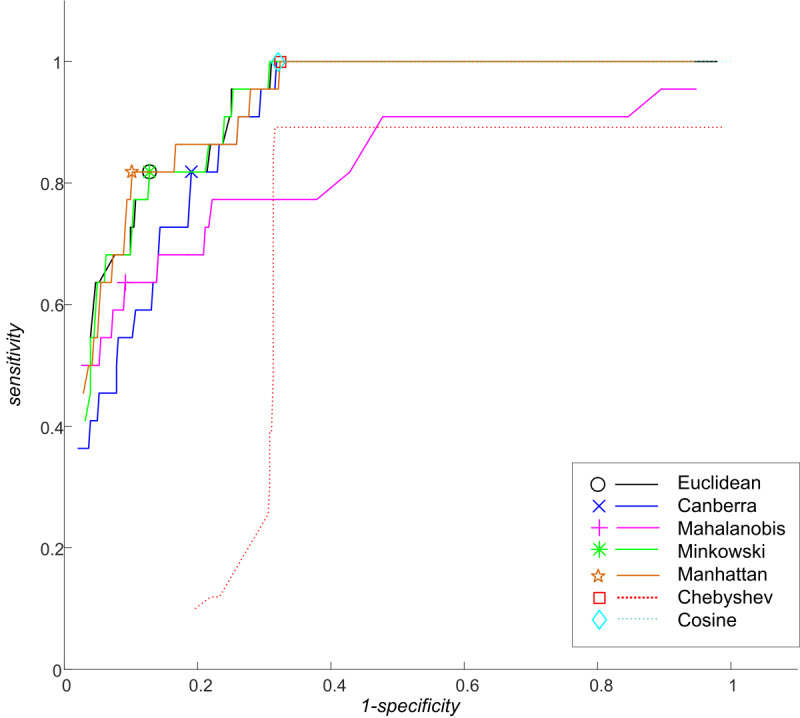
ROC curves generated for single distance metric-based classifiers. The curves were created by connecting 81 points showing the true positive rate (sensitivity) and the false positive rate (1-specificity) calculated at various threshold settings ranging from 5th percentile distance to 95th percentile distance. The highlighted points indicate the thresholds with the best achieved detection performance as determined by the criterion *C_1_*.

Figure 5 shows the ROC curves calculated for single-distance metric–based classifiers whose function was to find the optimal thresholds. The worst detection performances achieved by individual metrics were those of the Chebyshev and cosine metrics. The results were consistent for both data sets (see the lowest values of *C*_1_ in Table 3). These 2 distance metrics were, therefore, excluded from the subsequent ensemble classification. While a detailed ROC analysis was performed on the evaluation data set in order to find the best thresholds among 81 sampled and tested percentiles, in the case of the independent data set the performance characteristics were calculated only for 1 distance threshold setting.

Once the thresholds were set, the best combination of the 5 remaining distance metrics was searched for. All possible ensembles were generated, first employing the distance metric–based classifiers individually, then combining 2, 3, 4 of them and, finally, all 5 classifiers were combined in 3 different scenarios: (1) the thresholds were set using the evaluation data set and so was the best combination of metrics (Table 4); (2) the thresholds were set using the evaluation data set whereas the combination of metrics was searched for using the independent data set (Table 5); (3) the thresholds and the combination of metrics were searched for using the independent data set only (Table 6).

The second scenario proved best in mimicking the real use of the detection algorithm, which would be required to detect anomalies in yet unseen data. Specifically, the best detection performance was achieved using the combination of Mahalanobis, Manhattan, and Canberra distance metrics, resulting in sensitivity of 85.7%, specificity of 72.7%, and balanced accuracy of 79.2%.

As anticipated, higher performance rates were achieved when the data sets were used separately for threshold setting and for searching the best combination of the distance metrics—as indicated in scenarios (1) and (2).

**Table 3:** The characteristics of detection performance achieved by individual single-distance metric–based classifiers using the evaluation data set and the independent data set.^a^

Distance metric				Percentile threshold		Sensitivity (%)		Specificity (%)		Accuracy (%)		Youden index		ULC_dist		*C_1_*
**Evaluation data set (Registry number 3)**																
		Canberra	77.5		81.82		80.94		80.99		0.628		0.263		1.481	
		**Chebyshev**	**64.0**		**100.00**		**67.62**		**69.38**		**0.676**		**0.324**		**1.384**	
		**Cosine**	**95.0**		**100.00**		**67.89**		**69.63**		**0.679**		**0.321**		**1.395**	
		Euclidean	86.0		81.82		87.21		86.91		0.690		0.222		1.760	
		Mahalanobis	88.0		63.64		90.86		89.38		0.545		0.375		1.423	
		Manhattan	86.0		81.82		89.82		89.38		0.716		0.208		1.882	
		Minkowski	83.5		81.82		87.21		86.91		0.690		0.222		1.760	
**Independent data set (Registry number 5)**																
	Canberra		77.5		57.14		86.36		79.31		0.435		0.450		1.177	
	**Chebyshev**		**64.0**		**28.57**		**50.00**		**44.83**		–**0.214**		**0.872**		–**0.595**	
	**Cosine**		**95.0**		**100.00**		**13.64**		**34.48**		**0.136**		**0.864**		–**0.517**	
	Euclidean		86.0		42.86		90.91		79.31		0.338		0.579		0.916	
	Mahalanobis		88.0		28.57		90.91		75.86		0.195		0.720		0.465	
	Manhattan		86.0		42.86		95.46		82.76		0.383		0.573		1.084	
	Minkowski		83.5		42.86		90.91		79.31		0.338		0.579		0.916	

^a^The distance metrics with the lowest performance as determined by the criterion *C*_1_ (highlighted in bold) were excluded from the subsequent classification.

**Table 4:** The characteristics of detection performance achieved by various ensembles of distance metric–based classifiers using the evaluation data set only. Ten combinations with the highest performance as determined by the criterion *C^2^* are displayed.^a^

Combination of distance metrics	Sensitivity (%)	Specificity (%)	Balanced accuracy (%)	Error (%)	Precision (%)	*C_2_*
**MAN^b^** **, CAN^c^**	**95.46**	**82.51**	**88.98**	**16.79**	**23.86**	**1.844**
MAH^d^, CAN	95.46	81.98	88.72	17.28	23.33	1.842
EUC^e^, CAN	95.46	79.37	87.41	19.75	21.00	1.829
MINK^f^, CAN	95.46	79.37	87.41	19.75	21.00	1.829
EUC, MINK, CAN	95.46	79.37	87.41	19.75	21.00	1.829
EUC, MAN, CAN	95.46	78.85	87.15	20.25	20.59	1.826
MAN, MINK, CAN	95.46	78.85	87.15	20.25	20.59	1.826
EUC, MAN, MINK, CAN	95.46	78.85	87.15	20.25	20.59	1.826
MAH, MAN, CAN	95.46	76.50	85.98	22.47	18.92	1.814
EUC, MAH, CAN	95.46	74.15	84.80	24.69	17.50	1.803

^a^The best performing combination of the distance metrics is highlighted in bold.

^b^MAN: Manhattan.

^c^CAN: Canberra.

^d^MAH: Mahalanobis.

^e^EUC: Euclidean.

^f^MINK: Minkowski.

**Table 5:** The characteristics of detection performance achieved by various ensembles of distance metric–based classifiers using the evaluation data set and the independent data set. Ten combinations with the highest performance as determined by the criterion *C_2_* are displayed.^a^

Combination of distance metrics	Sensitivity (%)	Specificity (%)	Accuracy (%)	Balanced accuracy (%)	Error (%)	Precision (%)	*C_2_*
**MAH^b^** **, MAN^c^** **, CAN^d^**	**85.71**	**72.73**	**75.86**	**79.22**	**24.14**	**50.00**	**1.649**
CAN, EUC^e^, MAH, MAN, MINK^f^	85.71	68.18	72.41	76.95	27.59	46.15	1.627
EUC, MAH, CAN	85.71	68.18	72.41	76.95	27.59	46.15	1.627
MAH, MINK, CAN	85.71	68.18	72.41	76.95	27.59	46.15	1.627
EUC, MAH, MAN, CAN	85.71	68.18	72.41	76.95	27.59	46.15	1.627
EUC, MAH, MINK, CAN	85.71	68.18	72.41	76.95	27.59	46.15	1.627
MAH, MAN, MINK, CAN	85.71	68.18	72.41	76.95	27.59	46.15	1.627
MAH, MAN	71.43	86.36	82.76	78.90	17.24	62.50	1.503
EUC, MAH	71.43	81.82	79.31	76.62	20.69	55.56	1.481
MAH, MINK	71.43	81.82	79.31	76.62	20.69	55.56	1.481

^a^The best performing combination of the distance metrics is highlighted in bold.

^b^MAH: Mahalanobis.

^c^MAN: Manhattan.

^d^CAN: Canberra.

^e^EUC: Euclidean.

^f^MINK: Minkowski.

**Table 6:** The characteristics of detection performance achieved by various ensembles of distance metric–based classifiers using the independent data set only. Ten combinations with the highest performance as determined by the criterion *C_2_* are displayed.^a^

Combination of distance metrics	Sensitivity (%)	Specificity (%)	Accuracy (%)	Balanced accuracy (%)	Error (%)	Precision (%)	*C_2_*
**CAN^b^**	**85.71**	**86.36**	**86.21**	**86.04**	**13.79**	**66.67**	**1.718**
MAN^c^, CAN	85.71	81.82	82.76	83.77	17.24	60.00	1.695
EUC^d^, CAN	85.71	77.27	79.31	81.49	20.69	54.55	1.672
MINK^e^, CAN	85.71	77.27	79.31	81.49	20.69	54.55	1.672
EUC, MAN, CAN	85.71	77.27	79.31	81.49	20.69	54.55	1.672
EUC, MINK, CAN	85.71	77.27	79.31	81.49	20.69	54.55	1.672
MAN, MINK, CAN	85.71	77.27	79.31	81.49	20.69	54.55	1.672
EUC, MAN, MINK, CAN	85.71	77.27	79.31	81.49	20.69	54.55	1.672
MAH^f^, CAN	85.71	68.18	72.41	76.95	27.59	46.15	1.627
MAH, MAN, CAN	85.71	63.64	68.97	74.68	31.03	42.86	1.604

^a^The best performing combination of the distance metrics is highlighted in bold.

^b^CAN: Canberra.

^c^MAN: Manhattan.

^d^EUC: Euclidean.

^e^MINK: Minkowski.

^f^MAH: Mahalanobis.

## Discussion

### Anomaly Detection Context and Experiment Summary

In the era of EDC, it has become particularly difficult to process ever increasing data volumes in clinical registries. Data amount together with structural complexity of these databases make the task of anomaly detection, that may have a direct impact on the health care system, very demanding. Anomaly detection is an integral part of data analysis involving careful study of the identified anomalies and determination of their origin (data fraud, typing error, etc.), because it can significantly improve or negatively impact the subsequent analysis [19]. Even though anomalies tend to be misleading, they may carry valuable information [15,19]. For example, particular patient data may indicate that the patient has a different diagnosis than he/she is treated for, another anomalous pattern may indicate a new disease or reveal that investigators may have misinterpreted some questions. Therefore, detected anomalies need to be subjected to a careful assessment to mitigate the risk of losing valuable data by taking account of the unsuspicious ones, which may compromise the results and, as a consequence, lead to erroneous adjustments to clinical guidelines altering the current health care standards.

In this study, anomalous data were simulated and then detected. These operations were performed by a detection algorithm, whose detection performance was subsequently validated. The algorithm, running in a particular EDC system (Clade-IS), ends when automatic queries, whose function is to notify data managers and trial monitors of potentially anomalous data, have been generated. There are 2 key requirements which need to be met to implement such a detection algorithm in any EDC system successfully: (1) the ability of the system to create custom data views in the database and (2) the API able to react to data quality issues by its response (eg, a query generator). In the given settings, the accuracy was preferred over the algorithm execution time, so there was no need to optimize the algorithm for online use. A rapid online response is required when, for example, an intrusion activity is detected. This section presents a thorough description of (1) the detection algorithm running in the given EDC system and of (2) the actual validation experiments employing this algorithm together with the results interpretation. The findings are discussed here in terms of their validity and applicability (repeatability). The section is concluded with (3) a relevant literature review.

The tables loaded with raw data from 2 clinical registries were fed into the algorithm and a series of preprocessing steps, that is, dropping, imputation, recoding, and normalization, resulting in feature vectors were taken. These operations preceded the data simulation and the algorithm training. In the process, it was necessary to take account of data types which are supposed to be dropped as the given algorithm has not been devised to process all of them. Thus, some variables (texts, strings, and some raw JSON data) were excluded from further analysis. Although such an operation entails a significant information loss, it also represents a possible solution to the issues related to the “curse of dimensionality” (data reduction). One of the most difficult tasks was to handle the multiple instance forms supported by the Clade-IS. It means that the system allows not only forms limited to 1 instance per patient to be created, but also forms allowing more than 1 instance per patient. To tackle the problem of multiple forms filled in for a patient, the semiflattened tables have been introduced here. These tables aid in performing meaningful analysis and keep input data for each patient consistent, that is, with no blank attributes in places where data are expected. However, this approach has 2 limitations. First, the anomaly detection cannot be computed at the same time on all data available per patient. Instead, it is run separately on several semiflattened tables, each including data from 1 form structure instantiated multiple times. That said, anomalies resulting from a combination of forms with distinct form structures—the ones allowing multiple instances—could remain undetected. Second, information concerning data continuity (progression in time) that could possibly be filled in multiple forms created in a logical order was not investigated.

### Principal Results

The anomaly analysis was performed by calculating the distance between a centroid and data points using several distance metrics. There were 2 aspects assessed and recorded: (1) the Boolean identifier able to identify whether a patient is anomalous or not, and (2) the strength of anomaly evidence as determined by the number of distance metrics that labeled a patient as anomalous. The presented procedure could be potentially further improved using the medoid instead of the centroid. Medoids are robust cluster members that tend to be less sensitive to distant observations than averaged centroids are. When an anomaly is detected, the patient is labeled using automatically generated queries, which enable a person in charge to check this anomaly directly in a web application. Thus, the individual query may serve as an opportunity to implement appropriate corrective and preventive actions enhancing data integrity on the part of data managers and may also notify trial monitors of incorrect data entry in the initial phases of the study. Here, 2 neuroscience-related data sets were used for the algorithm validation; the first one served for training, thus setting the appropriate values for the algorithm parameters (distance metric thresholds); the second data set was used to validate the algorithm detection capability. It means that the preset detection algorithm was applied to the test data and its repeatability and applicability were investigated in practice. The percentile-based threshold could be set in 2 ways: (1) based on expert knowledge in the field and (2) setting the thresholds based on data. When percentage is defined by an expert, the number of expected anomalies to be detected is rather predictable and as such assists project managers in budget and staffing allocations, making the anomaly checks procedure more effective. The second, from our perspective a more sophisticated approach, was proposed and carried out in this study. Specifically, each distance metric threshold was identified using a combination of overall accuracy (the ratio between correctly classified anomalies and normal data) and measurements based on ROC curves (Youden index and curve distance from the upper-left corner). The optimal percentile threshold defined for each metric then varied from 77.5% to 95.0%. Therefore, the optimal number of patients to be investigated ranges between 5.0% and 22.5%, in order to uncover as many potential anomalies as possible while no time is wasted on checking normal data.

The experiment was performed on data set number 3, where the optimal thresholds were found, and data set number 5, which served for set up testing. The best result for data set number 3 employing a single distance metric was achieved by the Manhattan metric that labeled 14.0% (57/405) of patients as suspected to be anomalous: *C*_1_ (1.882), with sensitivity (81.8%) and specificity (89.8%) greater than 80.0%. When the thresholds (for each metric separately) were applied to the testing data set (number 5), the Canberra distance metric yielded the best results but sensitivity was very low compared with specificity: *C*_1_ of 1.177, sensitivity of 57.1%, and specificity of 86.4%. This suggests that, despite the high number of patients labeled as suspected to be anomalous (meeting the low percentile threshold, 77.5% in the case of Canberra), it is still not guaranteed that anomalous data will be detected. The other metrics had sensitivity or specificity below 50.0% and so we conclude that a single metric is insufficient to detect an anomaly.

Significantly better results were obtained when the distance metrics were combined. In this scenario, a patient, whose data were labeled as anomalous by at least one metric, was considered as suspected to be anomalous. This suggests that the proposed method reveals more suspicious data than methods based on single metrics. Sensitivity results for data set number 3 (shown in Table 5) were better than those obtained by any single metric alone (shown in Table 3). These results further suggest that combining 2 metrics can significantly outperform sensitivity of any single metric. Because none of the single metrics had sensitivity greater than 82.0%, it also suggests that the distance metrics complement each other when combined because they label different patients as anomalous. As the best results achieved by combining the metrics yielded the same sensitivity (95.5%), specificity had the decisive power when assessing the results. The best combination observed was that of the Manhattan distance metric and the Canberra distance metric, with specificity of 82.5%, accuracy of 83.2%, and *C*_2_ of 1.844. Combination of more than 2 metrics did not prove to be more efficient. In the case of validation data set number 5, the combination of 3 metrics (Mahalanobis, Manhattan, and Canberra) yielded the best results—sensitivity improved by almost 30% (85.7%), but specificity (approximately –14%; 72.7%) and overall accuracy (approximately –4%; 79.2%) were lower compared with the best single-metric performance (Canberra). These results also show that the threshold for the anomaly detection algorithm (method parameter), which has been set for 1 data set with a higher sample size (N), is possible to be applied to another data set, still producing satisfying results (Tables 4 and 5).

### Limitations

It needs to be noted that the proposed algorithm for anomaly detection is limited by the following: (1) clinical registries are frequently incomplete, with large amounts of missing data (the data sets studied here are not an exception). Because a significant number of incomplete variables were removed in the data preprocessing phase (method parameter set by data manager), some valuable information could have been lost; (2) only quantitative data (or recoded qualitative data) can be further analyzed by the algorithm; (3) the detection algorithm is still computationally intensive and requires long detection times despite the fact that a large number of unfilled and unanalyzable variables had been removed, together with 2 distance metrics (Chebyshev and cosine). The most time-consuming part of the algorithm run is data preprocessing which lasts tens of minutes. The detection itself then takes less than 10 seconds per tested registry. The preprocessing and analysis are run at regular intervals and are not directly linked to the data entry action. The time required to detect an outlier since its onset is dependent on the interval, which is implementation dependent and usually set to 24 hours; and (4) the algorithm was validated on artificially simulated anomalies. Had the anomalous data been generated by field experts, such an approach could have proved effective in terms of expert-provided knowledge that would have ensured authenticity of the anomalies, making the validation more natural.

### Comparison With Prior Work

There have been several research papers published on medical anomaly detection–related topics, as outlier detection has been widely applied in medical informatics for addressing different issues. According to the reviews, there are several detection techniques used in the field of medicine that can be divided into the following categories, listed in descending percentage order [15]: statistical (55.4%), clustering (15.2%), classification (12.5%), and nearest neighbor (ie, distance based, 8.9%), etc. As the numbers imply, statistic-based techniques tend to be used most frequently; however, it is well-known that the statistical assessment is not applicable to small sample sizes [20], therefore anomaly detection performed in small-scale studies or sites involving too few patients often leads to increasing the false-positive rate. For more reviews on anomaly detection in general, see [21] and for statistical monitoring process suggestions, we recommend [20]. That paper involved a multidisciplinary team of clinicians, statisticians, and data managers, who created a study-specific algorithm to flag the patients and sites with potentially fabricated data, which turned out to be fabricated and implanted in 7 sites, totaling 43 patients in 4 studies. Their algorithm for identifying sites with fabricated data achieved slightly lower results—except for 1 study, sensitivity and specificity were greater than 70%. In another research work [22], the authors combined k-means and isolation forest techniques, because the isolation forest–based methods are capable of finding anomalous patients that are not situated on the edge of a feature space. They, however, did not use ROC curves to define thresholds, but instead [23] split their data set into 2 subsets—first one consisted of only categorical variables and the second one of only continuous variables. This approach enabled them to work with each subset separately, searching (1) for infrequent category combinations in the subset with the categorical variables and (2) for distant objects defined by the cosine distance from the global mean in the subset with continuous variables. Then, they defined an anomaly score for each data object in both subsets. Adopting this approach, that is, splitting the data set into 2 subsets, could potentially improve our results. However, there would be many other parameters to be defined, such as the number of category combinations, that would complicate the setting of our anomaly detection algorithm. Estiri et al [24] used a different approach focusing on implausible rather than outlier data. The authors proposed a hierarchical k-means method to detect implausible observations, regardless of their values, that flag sparse clusters as anomalous, assuming no systematic errors. They also demonstrated that their clustering approach outperformed the conventional anomaly detection one that uses the standard deviation and Mahalanobis distance for identifying implausible laboratory data in the electronic health record. Although the authors consider the Mahalanobis distance to be standard, it did not work so well for us, especially in comparison with the other distance metrics (Table 3). To our knowledge, no paper presenting an EDC system with a built-in anomaly detection algorithm has been published to date.

### Conclusions

We have proposed and described an algorithm for detection of anomalous data in clinical registries, which has been implemented in a particular EDC system. The algorithm has proved to be capable of detecting anomalous data with sensitivity greater than 85%. Besides, the detection results were satisfactory for preset parameter settings derived from a different data set which enabled the algorithm to be applied in practice. In future work, we will inspect queries in real-world settings in order to assess precision and usefulness of the proposed anomaly detector from the viewpoint of data managers and also users with other roles, such as site monitors and clinical investigators. Other ideas for further research include an investigation into expert-generated anomalies and finding ways to speed up the detection algorithm.

## Appendix

Multimedia Appendix 1 Exemplification of the structure of patient A data (JSON).

Multimedia Appendix 2 Exemplification of the structure of patient B data (JSON).

## Abbreviations

CAN: Canberra metric
CHEB: Chebyshev metric
Clade-IS: Clinical Data Warehousing Information System
COS: cosine metric
CSM: central statistical monitoring
eCRF: electronic case report form
EAV: entity–attribute–value
EDC: electronic data capture
ETL: extract–transform–load
EUC: Euclidean metric
GDPR: General Data Protection Regulation
JSON: JavaScript object notation
MAH: Mahalanobis metric
MAN: Manhattan metric
MINK: Minkowski metric
REST API: representational state transfer application interface
ROC: receiver operating characteristic
RWD: real-world data

## References

[1] Solomon DJ, Henry RC, Hogan JG, Van Amburg GH, Taylor J (1991). Evaluation and implementation of public health registries. Public Health Rep.

[2] Hoque DME, Kumari V, Hoque M, Ruseckaite R, Romero L, Evans SM (2017). Impact of clinical registries on quality of patient care and clinical outcomes: A systematic review. PLoS One.

[3] Lu Z (2010). Technical challenges in designing post-marketing eCRFs to address clinical safety and pharmacovigilance needs. Contemp Clin Trials.

[4] Arts Danielle G T, De Keizer Nicolette F, Scheffer Gert-Jan (2002). Defining and improving data quality in medical registries: a literature review, case study, and generic framework. J Am Med Inform Assoc.

[5] O’Reilly GM, Gabbe B, Moore L, Cameron PA (2016). Classifying, measuring and improving the quality of data in trauma registries: A review of the literature. Injury.

[6] Houston L, Probst Y, Martin A (2018). Assessing data quality and the variability of source data verification auditing methods in clinical research settings. Journal of Biomedical Informatics.

[7] Timmermans C, Doffagne E, Venet D, Desmet L, Legrand C, Burzykowski T, Buyse M (2015). Statistical monitoring of data quality and consistency in the Stomach Cancer Adjuvant Multi-institutional Trial Group Trial. Gastric Cancer.

[8] George SL, Buyse M (2015). Data fraud in clinical trials. Clinical Investigation.

[9] Walther B, Hossin S, Townend J, Abernethy N, Parker D, Jeffries D (2011). Comparison of electronic data capture (EDC) with the standard data capture method for clinical trial data. PLoS One.

[10] van Dam J, Omondi Onyango K, Midamba B, Groosman N, Hooper N, Spector J, Pillai G(, Ogutu B (2017). Open-source mobile digital platform for clinical trial data collection in low-resource settings. BMJ Innov.

[11] Kaur S, Singh I, Gazali (2017). Artificial intelligence based clinical data management systems: A review. Informatics in Medicine Unlocked.

[12] Bruland P, Doods J, Brix T, Dugas M, Storck M (2018). Connecting healthcare and clinical research: Workflow optimizations through seamless integration of EHR, pseudonymization services and EDC systems. International Journal of Medical Informatics.

[13] Zhengwu Lu (2010). Electronic Data-Capturing Technology for Clinical Trials: Experience with a Global Postmarketing Study. IEEE Eng. Med. Biol. Mag.

[14] Brandt CA, Argraves S, Money R, Ananth G, Trocky NM, Nadkarni PM (2006). Informatics tools to improve clinical research study implementation. Contemporary Clinical Trials.

[15] Gaspar J, Catumbela E, Marques B, Freitas A (2011). Systematic review of outliers detection techniques in medical data - preliminary study. Proceedings of the International Conference on Health Informatics - Volume 1: HEALTHINF, (BIOSTEC 2011).

[16] Sakamoto J (2015). A Hercule Poirot of clinical research. Gastric Cancer.

[17] Lei D, Zhu Q, Chen J, Lin H, Yang P (2012). Automatic K-Means Clustering Algorithm for Outlier Detection. Information Engineering and Applications. Lecture Notes in Electrical Engineering.

[18] Youden WJ (1950). Index for rating diagnostic tests. Cancer.

[19] Smiti A (2020). When machine learning meets medical world: Current status and future challenges. Computer Science Review.

[20] Knepper D, Lindblad AS, Sharma G, Gensler GR, Manukyan Z, Matthews AG, Seifu Y (2016). Statistical Monitoring in Clinical Trials: Best Practices for Detecting Data Anomalies Suggestive of Fabrication or Misconduct. Ther Innov Regul Sci.

[21] Pimentel MA, Clifton DA, Clifton L, Tarassenko L (2014). A review of novelty detection. Signal Processing.

[22] Karczmarek P, Kiersztyn A, Pedrycz W, Al E (2020). K-Means-based isolation forest. Knowledge-Based Systems.

[23] Koufakou A, Georgiopoulos M (2009). A fast outlier detection strategy for distributed high-dimensional data sets with mixed attributes. Data Min Knowl Disc.

[24] Estiri H, Klann JG, Murphy SN (2019). A clustering approach for detecting implausible observation values in electronic health records data. BMC Med Inform Decis Mak.

