# Supplementary material for: Anomaly Detection Algorithm for Real-World Data and Evidence in Clinical Research: Implementation, Evaluation, and Validation Study
Source: JMIR Med Inform. 2021 May 7;9(5):e27172. doi: 10.2196/27172 (PMC8140384; doi:10.2196/27172)
Supplement: Multimedia Appendix 1 [file medinform_v9i5e27172_app1.pdf]

This file exemplifies the structure of a subject data (data belonging to one patient) stored in CLADE-IS EDC system (Registry No. 5).

This example subject A has 3 filled in forms of different type. One form equals one json below. Question legend can be found below.

NOTE: Sensitive data are anonymized (randomly replaced - eg. date of birth).

Form A: {"Q1":{"value":"EW","state":"done"},"Q2":{"value":103,"state":"done"},"Q3":{"value":"1984-10-21","state":"done"},"Q4":{"value":37,"state":"done"},"Q921":{"value":"29","state":"done"},"Q5":{"value":104,"state":"done"}}

Form B:
{"Q6":{"value":107,"state":"done"},"Q7":{"value":111,"state":"done"},"Q8":{"value":128,"state":"done"},"Q9":{"value":126,"state":"done"},"Q10":{"value":null,"state":"empty"},"Q977":{"value":104,"state":"done"},"Q13":{"value":"myopie, alergie pyl, prach, pe\u0159\u00ed, srst","state":"done"},"Q973":{"value":"neg","state":"done"},"Q974":{"value":"neg","state":"done"},"Q975":{"value":"matka i sestra zv\u00fd\u0161en\u00fd abusus alkoholu, sestra v AT ambulanci","state":"done"},"Q976":{"value":"neg","state":"done"},"Q14":{"value":"bpn","state":"done"},"Q15":{"value":"bpn","state":"done"},"Q16":{"value":null,"state":"empty"},"Q17":{"value":null,"state":"empty"},"Q18":{"value":null,"state":"empty"},"Q19":{"value":null,"state":"empty"},"Q922":{"value":"1967","state":"done"},"Q923":{"value":"1962","state":"done"},"Q978":{"value":"F200","state":"done"},"Q979":{"value":null,"state":"empty"},"Q980":{"value":null,"state":"empty"},"Q981":{"value":18,"state":"done"},"Q26":{"value":true,"state":"done"}}

Form C: {"Q914":{"value":"2000-01-16","state":"done"},"Q89":{"value":"tbl Zyprexa 10 mg 1-0-1 12 dn\u00ed","state":"done"},"Q90":{"value":1.78,"state":"done"},"Q91":{"value":56,"state":"done"},"Q92":{"value":17.7,"state":"done"},"Q93":{"value":"80","state":"done"},"Q94":{"value":85,"state":"done"},"Q95":{"value":"53","state":"done"},"Q96":{"value":"97","state":"done"},"Q97":{"value":20,"state":"done"},"Q98":{"value":2,"state":"done"},"Q99":{"value":32.7,"state":"done"},"Q100":{"value":"1345","state":"done"},"Q101":{"value":5.77,"state":"done"},"Q102":{"value":3.68,"state":"done"},"Q907":{"value":null,"state":"empty"},"Q103":{"value":null,"state":"skipped"},"Q104":{"value":0.26,"state":"done"},"Q105":{"value":0.68,"state":"done"},"Q106":{"value":0.71,"state":"done"},"Q594":{"value":0.65,"state":"done"},"Q107":{"value":null,"state":"empty"},"Q108":{"value":null,"state":"skipped"},"Q109":{"value":19.9,"state":"done"},"Q1042":{"value":null,"state":"empty"},"Q251":{"value":null,"state":"empty"},"Q111":{"value":4.3,"state":"done"},"Q112":{"value":31,"state":"done"},"Q113":{"value":3.7,"state":"done"},"Q595":{"value":0.64,"state":"done"},"Q597":{"value":1.6,"state":"done"},"Q598":{"value":1.8,"state":"done"},"Q983":{"value":2.1,"state":"done"},"Q1021":{"value":true,"state":"done"},"Q600":{"value":null,"state":"empty"},"Q601":{"value":0.67,"state":"done"},"Q1020":{"value":null,"state":"empty"},"Q114":{"value":0.15,"state":"done"},"Q115":{"value":3094,"state":"done"},"Q116":{"value":"322,8","state":"done"},"Q117":{"value":13.8,"state":"done"},"Q118":{"value":1.05,"state":"done"},"Q130":{"value":104.1,"state":"done"},"Q1017":{"value":true,"state":"done"},"Q602":{"value":null,"state":"empty"},"Q120":{"value":false,"state":"done"},"Q121":{"value":null,"state":"skipped"},"Q122":{"value":false,"state":"done"},"Q123":{"value":null,"state":"skipped"},"Q124":{"value":false,"state":"done"},"Q125":{"value":null,"state":"skipped"},"Q126":{"value":false,"state":"done"},"Q127":{"value":null,"state":"skipped"},"Q128":{"value":false,"state":"done"},"Q129":{"value":null

,"state":"skipped"},"Q131":{"value":386,"state":"done"},"Q133":{"value":"82","state":"done"},"Q28":{"value":5,"state":"done"},"Q924":{"value":"20","state":"done"},"Q925":{"value":"7","state":"done"},"Q926":{"value":"19","state":"done"},"Q1044":{"value":46,"state":"done"},"Q994":{"value":216,"state":"done"}}

Legend:

Form A

"Q4 = Age"

"Q3 = Date of birth"

"Q1 = Initials"

"Q921 = Age of admission"

"Q5 = Hand preference"

"Q2 = Gender"

Form B

"Q978 = ICD-10 Diagnosis"

"Q10 = Age Education"

"Q9 = Education"

"Q7 = Social Terms"

"Q974 = RAAf troubleshooting"

"Q980 = Psychiatric comorbidities"

"Q973 = RA psychozy"

"Q15 = PM development"

"Q6 = Family status"

"Q26 = MINI"

"Q19 = Psychiatry contact"

"Q922 = Mother details"

"Q975 = RA abusus"

"Q923 = Father details"

"Q14 = Circumstances of Child birth"

"Q17 = Current Pharmacotherapy"

"Q976 = Suicides"

"Q13 = Personal History"

"Q981 = Duration of untreated psychosis"

"Q977 = Hand Preferences"

"Q18 = Last Pharmacotherapy"

"Q16 = Childhood"

"Q979 = Diagnosis according to DSM-V"

"Q8 = Employment"

Form C

"Q116 = Kortisol"

"Q96 = Pulse"

"Q926 = G-PANSS"

"Q90 = Height"

"Q1020 = Impossibility to Determine Alldosterone"

"Q105 = Liver tests"

"Q1017 = Negative Cortisol"

"Q107 = Liver test patolpgy"

"Q598 = Lipid profile LDL"

"Q92 = BMI"

"Q101 = Leukocytes"

"Q93 = Dog"

"Q89 = Medication"

"Q1044 = PANSS score"

"Q983 = Lipid profile HDL"

"Q115 = Prolactin"

"Q114 = Aldosterone"

"Q102 = Neutrophils"

"Q97 = Fat Content"

"Q109 = Bilirubin"

"Q112 = GykovanyHb"

"Q108 = Liver tests patological values"

"Q594 = Liver tests ALP"

"Q1042 = Unmeasurable CDT"

"Q118 = TSH"

"Q133 = Pulse"

"Q111 = GLC"

"Q127 = Toxic. cocaine value"

"Q124 = Toxic. amphetamine"

"Q123 = Toxic. meatamphetamine value"

"Q128 = Toxic. Opiaty"

"Q126 = Toxic. ocaine"

"Q98 = Content of visceral fat"

"Q600 = Lipid ProfileP (a)"

"Q103 = PathologicalValues"

"Q595 = Lipid Profile TAG"

"Q1018 = CTD positive"

"Q99 = Muscle mass"

"Q121 = Toxic. cannabinoids value"

"Q117 = fT4"

"Q601 = Lipid profile ApoB"

"Q924 = P-PANSS"

"Q1019 = NegCTD"

"Q125 = ToxiAmphetamineValue"

"Q119 = ToxicologyPower"

"Q113 = Lipid ProfileChol"

"Q120 = Toxic. cannabinoids"

"Q100 = RMMet"

"Q925 = N-PANSS"

"Q597 = Lipid HDL Profile"

"Q914 = DateTyden0"

"Q95 = DiasPressure"

"Q994 = C-SSRS"

"Q122 = Toxic. metamphetamine"

"Q602 = DST Kortizol"

"Q94 = SysPress"

"Q131 = QTc"

"Q28 = CGI-S"

"Q907 = Blood patology"

"Q251 = CDT"

"Q1021 = Unable to determine LPa"

"Q106 = Liver tests AST"

"Q104 = Liver tests GGT"

"Q130 = DSTKortI"

"Q129 = Toxic. opiates value"

"Q91 = Weight"
