# Supplementary material for: Anomaly Detection Algorithm for Real-World Data and Evidence in Clinical Research: Implementation, Evaluation, and Validation Study
Source: JMIR Med Inform. 2021 May 7;9(5):e27172. doi: 10.2196/27172 (PMC8140384; doi:10.2196/27172)
Supplement: Multimedia Appendix 2 [file medinform_v9i5e27172_app2.pdf]

This file exemplifies the structure of a subject data (data belonging to one patient) stored in CLADE-IS EDC system.

This example subject B has 3 filled in forms of different type. One form equals one json below. Question legend can be found below.

NOTE: Sensitive data are anonymized (randomly replaced - eg. birth year).

Form A:
{"Q1":{"value":"FR","state":"done"},"Q2":{"value":100,"state":"done"},"Q3":{"value":"1966","state":"done"},"Q4":{"value":53,"state":"done"},"Q5":{"value":103,"state":"done"},"Q6":{"value":"TXTT","state":"done"},"Q9":{"value":null,"state":"skipped"},"Q10":{"value":null,"state":"skipped"}}

Form B:
{"Q143":{"value":true,"state":"done"},"Q144":{"value":true,"state":"done"},"Q145":{"value":true,"state":"done"},"Q146":{"value":true,"state":"done"},"Q147":{"value":"2017-11-03","state":"done"},"Q149":{"value":null,"state":"empty"},"Q151":{"value":null,"state":"empty"},"Q152":{"value":null,"state":"empty"},"Q153":{"value":null,"state":"empty"},"Q154":{"value":null,"state":"empty"},"Q155":{"value":null,"state":"empty"},"Q156":{"value":null,"state":"empty"}}"

Form C:
{"Q13":{"value":null,"state":"empty"},"Q14":{"value":null,"state":"empty"},"Q15":{"value":null,"state":"empty"},"Q2462":{"value":null,"state":"empty"},"Q2463":{"value":null,"state":"skipped"},"Q16":{"value":"12","state":"done"},"Q17":{"value":111,"state":"done"},"Q3202":{"value":null,"state":"empty"},"Q3203":{"value":null,"state":"empty"},"Q3204":{"value":null,"state":"empty"},"Q3205":{"value":null,"state":"empty"},"Q3206":{"value":true,"state":"done"},"Q3207":{"value":null,"state":"empty"},"Q19":{"value":125,"state":"done"},"Q20":{"value":137,"state":"done"},"Q21":{"value":142,"state":"done"},"Q2831":{"value":null,"state":"empty"},"Q22":{"value":148,"state":"done"},"G155":[],"G156":[{"Q1955":{"value":"cysteklomie na koleni","state":"done"},"Q1956":{"value":"1983","state":"done"},"id":1},{"Q1955":{"value":"tonselektomie","state":"done"},"Q1956":{"value":"1983","state":"done"},"id":2}],"G331":[],"G157":[]}

Legend:

Form A

"Q10 ="; "Phone"

"Q9 = Email"

"Q1 = Initials"

"Q5 = Hand Preferences"

"Q4 = Age"

"Q2 = Gender"

"Q6 = Code"

"Q3 = Birth year"

Form B

"Q155 = Contraindications MRI"

"Q142 = Input criteria filled"

"Q154 = Brain examination Found"

"Q150 = Exclusion criteria filled"

"Q152 = Diagnosis of relative"

"Q145 = Age"

"Q144 = No psychiatric diagnosis"

"Q153 = Organic Fault"

"Q146 = InformedSent"

"Q143 = M.I.N.I."

"Q149 = Psychiatric diagnosis"

"Q147 = Date"

"Q156 = Pregnancy"

"Q151 = Cognitive Deficiency"

Form C

"Q3205 = Learning disorders"

"Q3203 = Faults repeated years"

"Q3202 = Attention defects"

"Q1956 = Operation year"

"Q3041 = Level 1"

"Q2831 = Current receipt"

"Q3207 = Faults not known"

"Q3040 = Davka 1"

"Q15 = Encephalitis meningitis"

"Q12 = Specified unicked"

"Q1958 = Diagnosis"

"Q19 = Employed"

"Q1953 = Diseased"

"Q2462 = Ethnicity"

"Q21 = Housing"

"Q14 = Captured disabled"

"Q17 = Highest educated"

"Q22 = Financial position"

"Q20 = Family status"

"Q13 = Head injury"

"Q3206 = Rear Faults"

"Q3204 = Special education disorders"

"Q2463 = Other ethnicity"

"Q18 = Learning Disorders"

"Q1957 = Related"

"Q1955 = Work"

"Q16 = Numer of school years"

"Q3039 = Medication 1"

"Q1954 = Illness"
